# Molecular Cloning and Expression of *MnGST-1* and *MnGST-2* from Oriental River Prawn, *Macrobrachium nipponense*, in Response to Hypoxia and Reoxygenation

**DOI:** 10.3390/ijms19103102

**Published:** 2018-10-10

**Authors:** Lei Xu, Ming Yang, Hongtuo Fu, Shengming Sun, Hui Qiao, Wenyi Zhang, Yongsheng Gong, Sufei Jiang, Yiwei Xiong, Shubo Jin, Yan Wu

**Affiliations:** 1 Wuxi Fishery College, Nanjing Agricultural University, Wuxi 214081, China; xwmr258@163.com (L.X.); 18931340665@163.com (M.Y.); 2 Key Laboratory of Freshwater Fisheries and Germplasm Resources Utilization, Ministry of Agriculture, Freshwater Fisheries Research Center, Chinese Academy of Fishery Sciences, Wuxi 214081, China; sunsm@ffrc.cn (S.S.); qiaoh@ffrc.cn (H.Q.); zhangwy@ffrc.cn (W.Z.); gongys@ffrc.cn (Y.G.); jiangsf@ffrc.cn (S.J.); xiongyw@ffrc.cn (Y.X.); jinsb@ffrc.cn (S.J.); wuy@ffrc.cn (Y.W.)

**Keywords:** *Macrobrachium nipponense*, hypoxia, reoxygenation, mRNA expression, enzyme activity

## Abstract

The glutathione-S-transferase (GST) superfamily includes seven classes, and different classes have different functions. GST superfamily members function in various processes including detoxification of xenobiotics, protection against oxidative damage, and intracellular transport of hormones, endogenous metabolites, and exogenous chemicals. Herein, to elucidate the tissue-specific expression pattern of GSTs in response to hypoxia stress, which induces cell death, we investigated the expression of GSTs in response to hypoxia and reoxygenation in oriental river prawn, *Macrobrachium nipponense*. Full-length cDNAs of two δ class GSTs were cloned from the hepatopancreas, and named *MnGST-1* and *MnGST-2* based on the established GST nomenclature system. Expression profiles of both GSTs in various tissues were different under acute and chronic experimental hypoxia stress conditions, suggesting that both respond strongly to hypoxia-induced oxidative stress. However, the intensity of responses to hypoxia and reoxygenation were different in different tissues. During acute hypoxia stress, *MnGST-1* responds earlier than *MnGST-2* in the hepatopancreas and gill, but more slowly in muscle. By contrast, during chronic hypoxia stress, *MnGST-2* plays a more important role in the hepatopancreas and gill than *MnGST-1*.

## 1. Introduction

The level of dissolved oxygen (DO) is a major indicator of water quality, particularly in the prawn farming industry. ‘Dead zones’ is the name given to areas in coastal waters in which biodiversity is obliterated due to the detrimental effects of low DO [1,2,3,4]. Under hypoxia conditions, accumulated electrons contribute to the formation of damaging reactive oxygen species (ROS) [5], and reoxygenation of hypoxic tissues can lead to ROS generation [6]. ROS damage important biomolecules including DNA, proteins and lipids. Apoptosis is induced in cells excessively damaged by acute hypoxia, including tissues of shrimp gill [7]. To protect themselves against damage by ROS, aerobic organisms have evolved a set of antioxidant defense systems, including antioxidant enzymes, such as superoxide dismutase (SOD), catalase (CAT), glutathione peroxidase (GPx), and glutathione S-transferase (GST) [8,9,10,11].

The GST antioxidant system plays a fundamental role in cellular defense against reactive free radicals and other oxidants [12,13]. The GST protein superfamily are involved in various functions including detoxification of xenobiotics, protection against oxidative damage, and intracellular transport of hormones, endogenous metabolites and exogenous chemicals, in a wide variety of organisms [14,15,16,17]. Based on sequence similarity, immunological characteristics, kinetic properties, and tertiary structure, mammalian GSTs have been divided into eight groups; Alpha, Mu, Pi, Sigma, Theta, Delta, Kappa, and microsomal [18]. In insects, seven groups of GSTs have been identified [19]. Delta and Epsilon groups are only present in insect species, and Omega, Theta, Sigma, and Zeta, while microsomal groups are also present in other species. The GSTs that have been well characterized in *Venerupis philippinarum* are belong to Omega and Sigma class [20], while the GST that has been characterized in *Litopenaeus vannamei* belongs to the Mu class [17]. However, GSTs in oriental river prawn, *Macrobrachium nipponense*, have not been investigated. In goldfish, *Carassius auratus*, the enzyme activity of GSTs and other antioxidants must be controlled to ensure that oxyradical-induced lipid peroxidation does not exceed physiologically tolerable levels [21].

*M. nipponense* is an important commercial prawn species that is widely distributed in freshwater and low-salinity estuarine regions in China and other Asian countries. In China, it is one of the most important aquaculture species. Compared with most other crustaceans, *M. nipponense* is more sensitive to hypoxia [7,22,23]. Therefore, understanding the molecular mechanisms underpinning the stress responses to hypoxia and reoxygenation in *M. nipponense* is essential for the sustained development of prawn aquaculture. In the present study, we cloned and characterized full-length MnGST-1 and MnGST-2 cDNAs from *M. nipponense*, and quantified their tissue-specific mRNA expression levels prawns in response to hypoxia and reoxygenation. We also assayed the enzyme activity of the MnGST-1 and MnGST-2 proteins in prawns under hypoxia and reoxygenation conditions.

## 2. Results

### 2.1. Prawn MnGST-1 and MnGST-2 Coding Sequences

**Figure A1:**
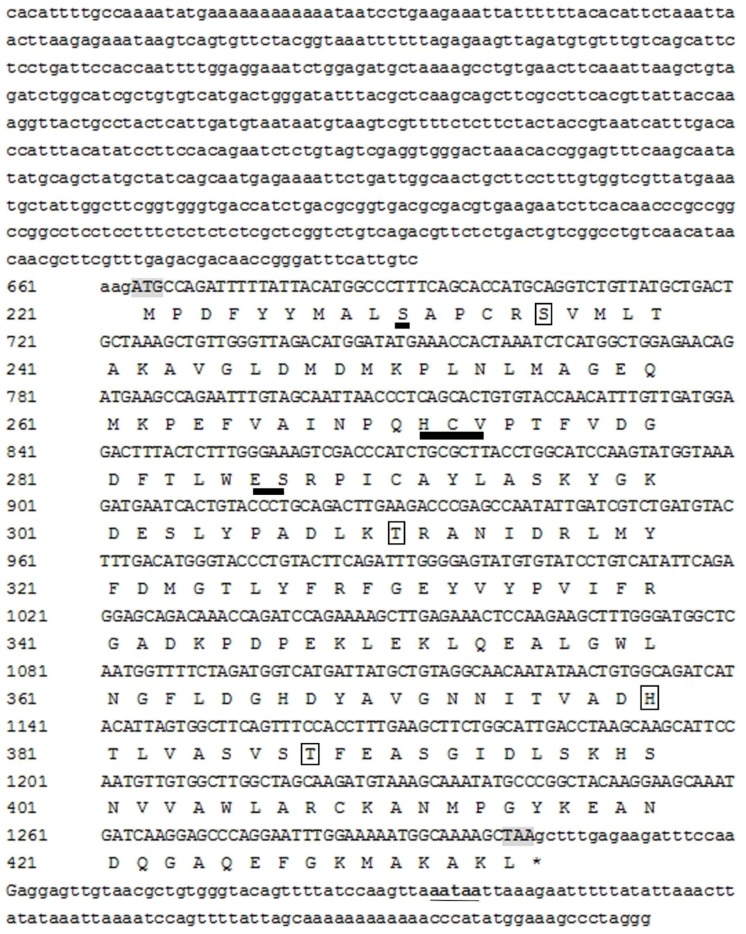
Nucleotide and predicted amino acid sequences of *MnGST-1* from oriental river prawn, *Macrobrachium nipponense*. Amino acid sequences are shown as capital letters below the nucleotide sequences. Black rectangles indicate the four potential O-GlcNAc sites. Black underlines indicate highly-conserved residues. The start codon (ATG) is boxed. The Stop codon (TAA) is shaded in grey. The protein translation terminator was denoted with an asterisk. The AATAA sequence is a potential polyadenylation signal, represented by an underscore.

**Figure A2:**
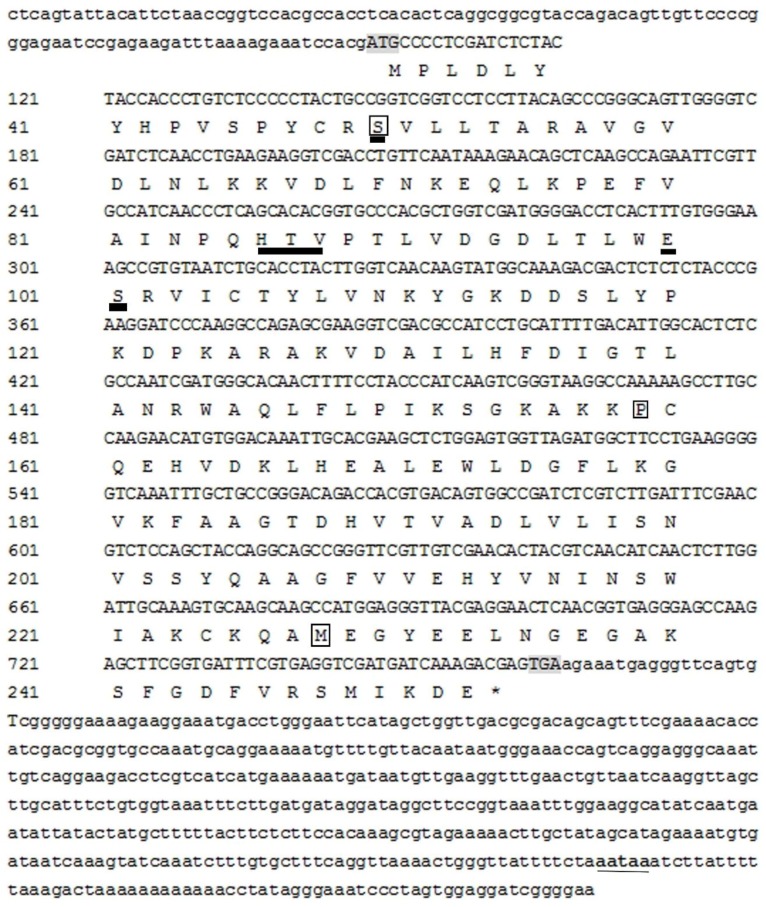
Nucleotide and predicted amino acid sequences of *MnGST-2*. Amino acid sequences are shown as capital letters below the nucleotide sequences. Black rectangles indicate the three potential O-GlcNAc sites. Black underlines indicate highly conserved residues. The start codon (ATG) is boxed. The stop codon (TGA) is shaded in grey. The protein translation terminator was denoted with an asterisk. The AATAA sequence is a potential polyadenylation signal, represented by an underscore.

Prawn *MnGST-1* comprises 1451 nucleotides, encoding a 214 amino acid polypeptide with a probable signal peptide cleavage site between the Gly24 and Leu25 amino acids. The deduced protein has a theoretical pI of 5.39 and an estimated molecular mass of 23.91 kDa. Start and stop codons are at positions 664 and 1308. The 5′-untranslated region (UTR) is 663 bp long, and the 3′-UTR is 143 bp long, including the poly-A tail (Figure A1). *MnGST-2* comprises 1248 nucleotides, encoding a 219 amino acid polypeptide with a probable signal peptide cleavage site between the Gly25 and Val26 amino acids. This deduced protein has a theoretical pI of 6.38 and an estimated molecular mass of 24.58 kDa. Start and stop codons are at positions 103 and 762. The 5′-UTR is 102 bp long, and the 3′-UTR is 486 bp long, including the poly-A tail (Figure A2). Four potential O-GlcNAc sites in *MnGST-1* (Ser15, Ser90, Ser160 and Ser167) and three potential O-GlcNAc sites in *MnGST-2* (Ser11, Ser119, and Ser168) were predicted by the YinOYang program. The N-terminal domain has six highly conserved residues (G-sites; Ser11, 52HTV54, and 66ES67), which are characteristic of the GST δ family. All primers used for cloning are listed in Table 1.

### 2.2. Comparison and Phylogenetic Analysis of M. nipponense GSTs with Other GSTs

**Figure 1:**
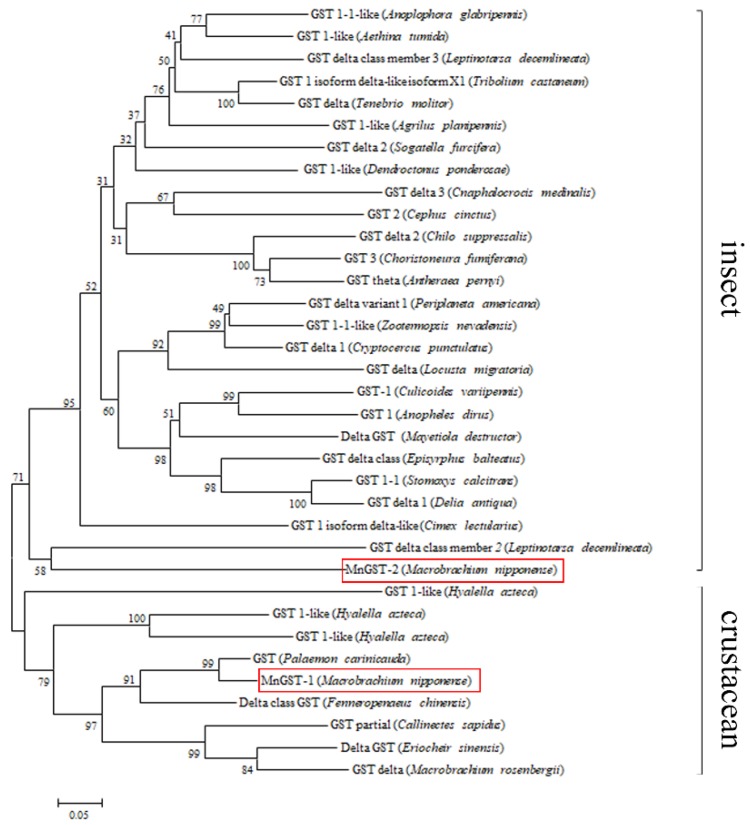
Phylogenetic tree based on GST sequences generated using the neighbor-joining method in the MEGA 5.10 program with 1000 bootstrap replicates. *MnGST-1* and *MnGST-2* were highlighted in red boxes.

**Figure A3:**
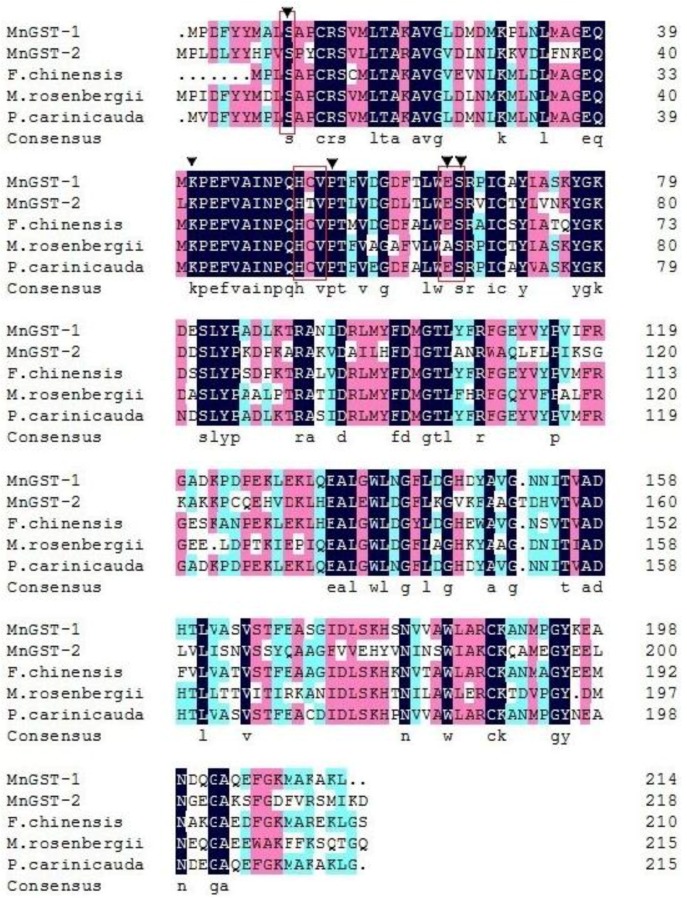
Multiple sequence alignment of the deduced amino acid sequences of *Macrobrachium nipponense* GSTs with those of other known GSTs. *MnGST-1* (GenBank MG787172), *MnGST-2* (GenBank MG787173), *Fenneropenaeus chinensis* (GenBank ANH58178.1), *M. rosenbergii* (GenBank CCQ19301.1), and *Palaemon carinicauda* (GenBank AGZ89666.1). Red rectangles indicate N-terminal serines. Black triangles indicate the conserved G-site.

BLAST (http://blast.ncbi.nim.nih.gov/Blast.cgi) and phylogenetic analysis of GSTs from *M. nipponense* confirmed that they belong to the GST δ class. As the first GSTs reported from *M. nipponense*, they were named *MnGST-1* and *MnGST-2*. Most of the deduced amino acid residues in the cDNAs were variable, but 75 amino acids were conserved in all GSTs (Figure A3). Phylogenetic tree analysis showed that *MnGST-1* is most closely related to a GST in *Palaemon carinicauda* (GenBank AGZ89666.1), while *MnGST-2* is most closely related to GST δ class member 2 of *Leptinotarsa decemlineata* (GenBank XP_023021064.1) (Figure 1). GenBank accession numbers are listed in Table 2.

### 2.3. Tissue Distribution of MnGST-1 and MnGST-2

**Figure 2:**
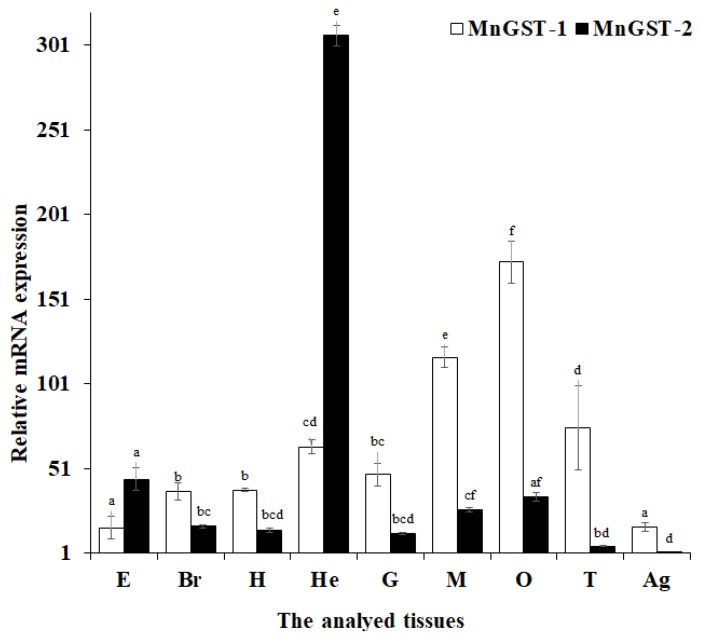
Expression of *MnGST-1* and *MnGST-2* in different tissues (E: eye; Br: brain; H: heart; He: hepatopancreas; G: gill; M: muscle; O: ovary; T: testis; Ag: abdominal ganglion) of *Macrobrachium nipponense.* Bar charts with different lowercase letters show significant differences in *MnGST-1* and *MnGST-2* (*p* < 0.05). Values are means ± standard error of the mean (SE) for triplicate samples.

QPCR (Quantitative real-time reverse transcription PCR) was used to quantify *MnGST-1* and *MnGST-2* mRNA expression levels in different tissues, and both *MnGST-1* and *MnGST-2* were detected in all tissues tested. *MnGST-1* mRNA levels were high in the hepatopancreas, gill, muscle, ovary, and abdominal ganglion. Highest expression in the ovary indicates that *MnGST-1* may be related to reproduction in female *M. nipponense* [11,24]. High-level expression of *MnGST-2* was observed in the eye, brain, heart, hepatopancreas, gill, muscle and ovary. Highest *MnGST-2* expression in the hepatopancreas may indicate a role in detoxification and protection against oxidative damage (Figure 2). Based in these expression results, hepatopancreas, gill, and muscle tissue were chosen for subsequent experiments.

### 2.4. Expression and Enzyme Activity of MnGST-1 and MnGST-2 in M. nipponense in Response to Acute Hypoxia and Reoxygenation

**Figure 3:**
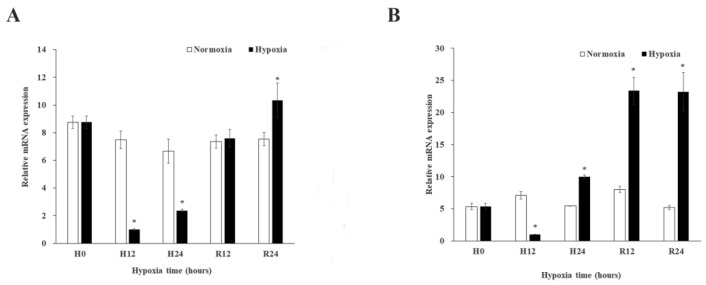
Expression of *MnGST-1* (**A**) and *MnGST-2* (**B**) in the hepatopancreas of *M. nipponense* at five time points after hypoxia and reoxygenation (H0: hypoxia for 0 h; H12: hypoxia for 12 h; H24: hypoxia for 24 h; R12: reoxygenation for 12 h; R24: reoxygenation for 24 h). Data indicated with asterisks are significantly different (*p* < 0.05) between treatment and control groups. Values are means ± SE for triplicate samples.

**Figure 4:**
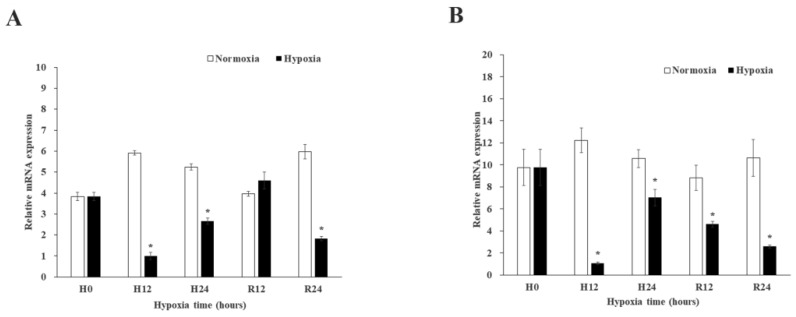
Expression of *MnGST-1* (**A**) and *MnGST-2* (**B**) in the gill of *M. nipponense* at five time points after hypoxia and reoxygenation (H0: hypoxia for 0 h; H12: hypoxia for 12 h; H24: hypoxia for 24 h; R12: reoxygenation for 12 h; R24: reoxygenation for 24 h). Data indicated with asterisks are significantly different (*p* < 0.05) between treatment and control groups. Values are means ± SE for triplicate samples.

**Figure 5:**
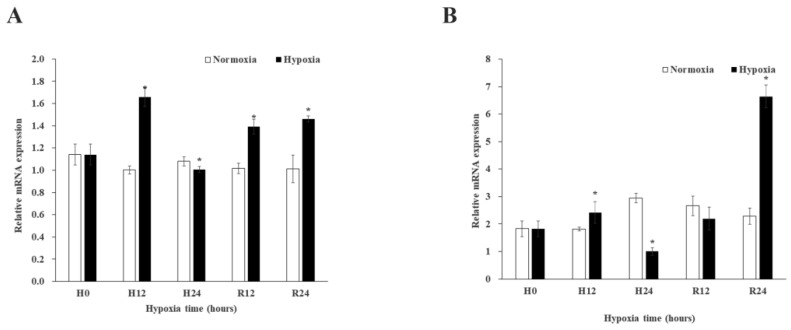
Expression of *MnGST-1* (**A**) and *MnGST-2* (**B**) in the muscle of *M. nipponense* at five time points after hypoxia and reoxygenation (H0: hypoxia for 0 h; H12: hypoxia for 12 h; H24: hypoxia for 24 h; R12: reoxygenation for 12 h; R24: reoxygenation for 24 h). Data indicated with asterisks are significantly different (*p* < 0.05) between treatment and control groups. Values are means ± SE for triplicate samples.

Since the results of QPCR can be supported by semi-quantitative analysis, the expression patterns of *MnGST-1* and *MnGST-2* were determined. Following exposure to hypoxia, expression of *MnGST-1* and *MnGST-2* in the hepatopancreas was minimal after 12 h, but increased thereafter. The expression levels in the hypoxia group were significantly different from those in the normal group at 0 h and after reoxygenation for 12 h (*p* < 0.05) (Figure 3A). This trend was the same for *MnGST-2* (Figure 3B). Under reoxygenation conditions, *MnGST-1* expression returned to normal levels, but this was not the case for *MnGST-2* (Figure 3). Furthermore, the situation was different between hepatopancreas and gill after reoxygenation. In the gill, *MnGST-1* expression was decreased significantly in the hypoxia group at 12 h (*p* < 0.05; Figure 4A). However, *MnGST-2* expression increased earlier, but was then decreased at 24 h in the hypoxia group compared with the control group (*p* < 0.05; Figure 4B). In muscle, expression of *MnGST-1* peaked at 12 h (Figure 5A), and the situation was similar for *MnGST-2*, although the peak was reached after reoxygenation for 24 h (Figure 5B).

**Figure 6:**
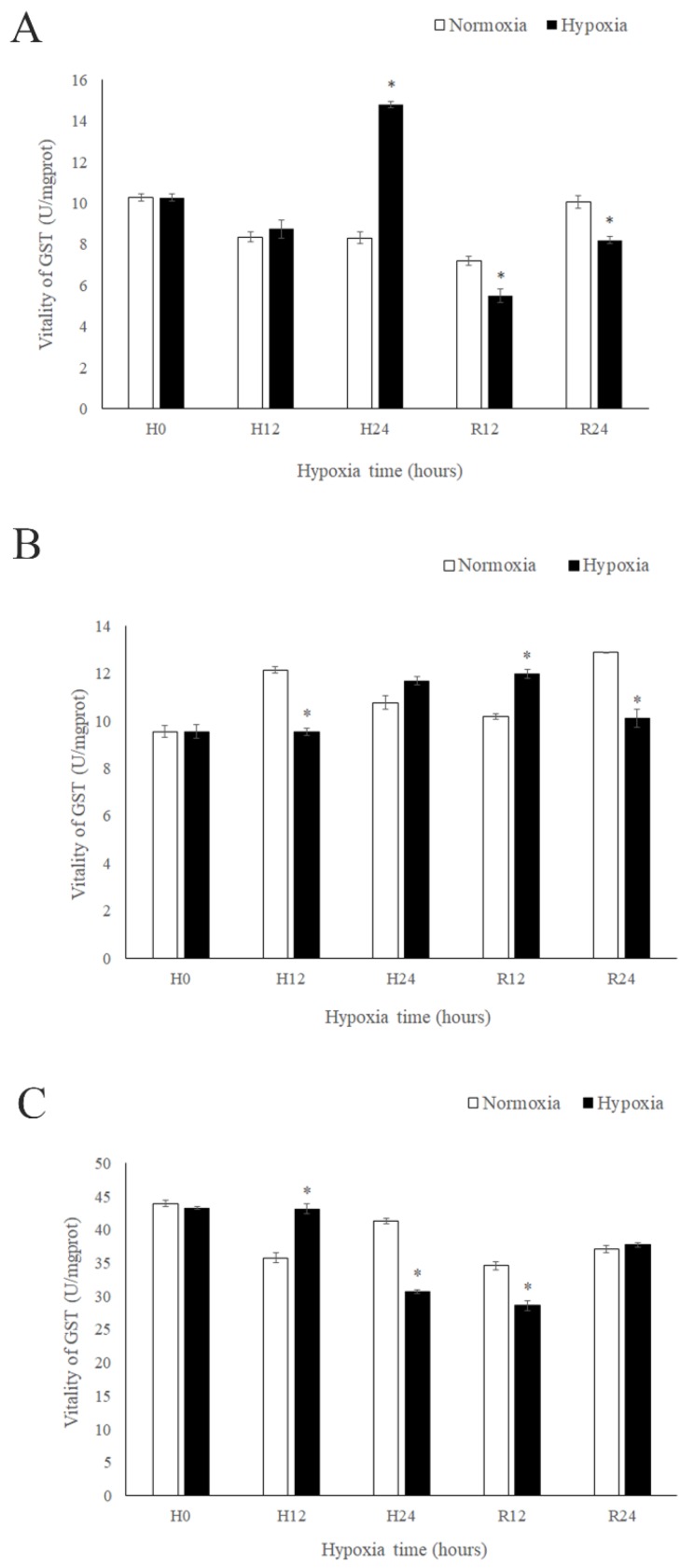
Enzyme activity of GSTs in the hepatopancreas (**A**), gill (**B**) and muscle (**C**) of *M. nipponense* at five time points after hypoxia and reoxygenatio (H0: hypoxia for 0 h; H12: hypoxia for 12 h; H24: hypoxia for 24 h; R12: reoxygenation for 12 h; R24: reoxygenation for 24 h). Data indicated with asterisks are significantly different (*p* < 0.05) between treatment and control groups. Values are means ± SE for triplicate samples.

The enzyme activity of GSTs was high 24 h after hypoxia treatment (Figure 6A). The enzyme activity of GSTs in gill initially increased then decreased following both hypoxia and reoxygenation (Figure 6B). The trends in the expression and enzyme activity of GSTs in the gill were the same as those in the hepatopancreas, and the trends in enzyme activity and expression of *MnGST-1* and *MnGST-2* were the same in muscle (Figure 6C), indicating that both *MnGST-1* and *MnGST-2* play an important role in this tissue during both hypoxic stress and reoxygenation.

### 2.5. Expression and Enzyme Activity of MnGST-1 and MnGST-2 in M. nipponense in Response to Chronic Hypoxia Stress

**Figure 7:**
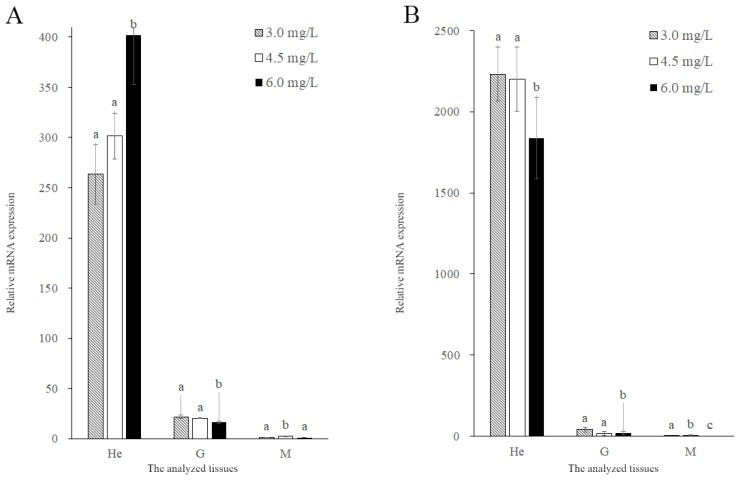
Expression of *MnGST-1* (**A**) and *MnGST-2* (**B**) in the hepatopancreas (He), gill (G) and muscle (M) of *M. nipponense* in response to chronic hypoxia stress experiment. Values are means ± SE for triplicate samples. Bars with different letters indicate significant differences (*p* < 0.05).

MnGST-1 expression and enzyme activity followed the same trend in gill tissue, with no significant differences between the two hypoxia groups, but there were significant differences between hypoxia groups and normal groups (*p* < 0.05). This trend was not observed for MnGST-1 expression in muscle (Figure 7A). For MnGST-2, there were no significant differences between the moderate hypoxia group and the normal group in the hepatopancreas and gill, but there were significant differences between the severe hypoxia group and the normal group (*p* < 0.05). The trend in expression in muscle was the same as that observed for MnGST-1 (Figure 7B).

**Figure 8:**
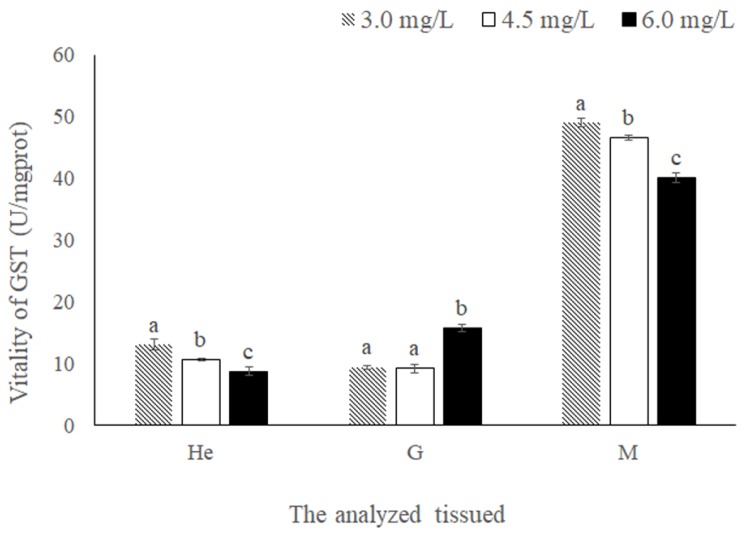
Enzyme activity of GSTs in the hepatopancreas (He), gill (G) and muscle (M) of *M. nipponense* in response to chronic hypoxia stress experiment. Data indicated with different letters are significantly different (*p* < 0.05) between treatment and control groups. Values are means ± SE for triplicate samples.

In hepatopancreas, the enzyme activity was increased with reduction of DO. In the gill, the enzyme activity of was decreased in hypoxic treatment groups (Figure 8). Following chronic hypoxia stress, GST enzyme activity coincided with MnGST-2 expression only in the hepatopancreas.

## 3. Discussion

We report here the cloning and expression of two GST subunits from the oriental river prawn Macrobrachium nipponense in response to hypoxia and reoxygenation. According to sequence alignment results, the N-terminal domains of both MnGST-1 and MnGST-2 include a highly-conserved serine residue at position 11 that plays an important role in the catalytic mechanism of GSTs [25]. Furthermore, both MnGST-1 and MnGST-2 include two serines, a lysine, a proline, and a glutamate that are located in the reduced glutathione (GSH) binding site of GSTs [25,26,27]. Functional domain analysis indicated that both prawn enzymes belong to the GST δ class, and based on the established GST nomenclature system, they were named MnGST-1 and MnGST-2, respectively. To date, all available GSTs have been classified into 13 different classes according to sequence homology and molecular characteristics, and some are species-specific [16,28,29]. Two GSTs described in the present study were classified as δ class enzymes based on the phylogenetic tree. Delta class GSTs were widely believed to be insect-specific, and this may need to be reconsidered. Theta class GSTs can appear in phylogenetic trees of δ class GSTs because the theta class is an ancient class among all GST families and includes a large group of subclasses and isoforms present in various species, whereas δ GSTs evolved later [29,30].

In this study, expression of both MnGST-1 and MnGST-2 decreased in the hepatopancreas and gill under hypoxia (Figure 3 and Figure 4). As previously reported [31], expression levels of mRNA for Crustacean Cardioactive Peptide in *M. nipponense* have the same trend in eyestalk tissue under hypoxia. In response to hypoxia, animals downregulate metabolism in order to increase survival [32,33]. Expression of both MnGST-1 and MnGST-2 was higher after reoxygenation than in the control group in hepatopancreas and muscle (Figure 3 and Figure 5), indicating that gene expression may be stimulated by both hypoxic stress and reoxygenation. Expression of MnGST-1 and MnGST-2 in the gill did not return to normal levels after reoxygenation (Figure 4), suggesting that the negative effects of hypoxia could not be completely reversed over the relatively short duration of the experiment. Furthermore, a continuing downward trend after reoxygenation suggests that tissues are injured following hypoxia [5,21,34,35,36,37]. These findings suggest that ROS are also generated during reoxygenation. Indeed, the sudden input of oxygen after environmental hypoxia increases ROS production in *Litopenaeus vannamei* [38]. By contrast, GST mRNA expression levels in muscle tissue of *Mytilus galloprovincialis* showed no significant changes after hypoxia stress for 24 h [39]. This apparent discrepancy may be due to differences between species or experimental conditions. In hepatopancreas and gill, MnGST-2 responds earlier than MnGST-1, suggesting that MnGST-2 is more sensitive to reoxygenation than MnGST-1. These results indicate that MnGST-1 and MnGST-2 might participate in distinct physiological processes in different tissues.

GST enzyme activity is an important indicator of damage to hepatopancreas tissue. In the present work, the enzyme activity reached a peak 24 h after hypoxia, indicating that damage to the hepatopancreas was maximal at 24 h. This is consistent with previous reports on *Chironomus riparius* [40] but different from *Leporinus macrocephalus* [41], possibly because prawns are more closely related to insects than fish. The trend in enzyme activity of GSTs was opposite to the trend of MnGST-1 and MnGST-2 expression from 0 to 24 h, suggesting these two GST subtypes do not play an important role in the hepatopancreas in response to acute hypoxic stress. However, the trends in enzyme activity and expression were the same for MnGST-1 during reoxygenation, suggesting that MnGST-1 plays a part in recovery from hepatopancreas injury. Both enzymes were expressed at the lowest levels at 12 h in the hepatopancreas and gill, but the lowest level in muscle occurred at 24 h, suggesting that muscle is a tissue that responds relatively late during the process of hypoxia. The trend of enzyme activity in muscle is consistent with results in *Pelteobagrus vachellin* [42]. However, in Carassius auratus, enzyme activity in muscle showed no significant differences between hypoxia and normal groups, but it was significantly increased after reoxygenation [21], and in *Mytilus galloprovincialis*, this trend was observed, albeit later [39], possibly due to species-specific differences. In *Macrobrachium malcolmsonii*, the enzyme activity in muscles increased significantly when it is exposed to endosulfan for 21 days. This situation might be caused by the different stress conditions [43]. The results showed that GST protein expression lagged somewhat behind mRNA expression, suggesting that the same genes/proteins may perform different functions in different tissues. Comparing enzyme activity in different tissues revealed that activity in muscle returned to normal levels after reoxygenation for 24 h, but this was not the case for hepatopancreas and gill, indicating that recovery from hypoxia varies in different tissues.

In chronic hypoxia experiments, the highest expression levels in the hepatopancreas implicate this as one of the most important antioxidant organs. Results in the gill were similar to those rats, in which expression of vascular endothelial growth factor was increased after chronic hypoxia [44]. The observed differences between the two GSTs indicates that MnGST-1 is more sensitive to hypoxia in the gill, consistent with the results of tissue distribution analysis (Figure 2). After prawns were exposed to hypoxia, GST enzyme activity in the hepatopancreas and muscle increased with decreasing DO concentration, consistent with the hepatopancreas of *Penaeus aztecus* [45]. GST enzyme activity coincided with MnGST-2 expression only in the hepatopancreas, indicating that MnGST-2 may have an important role in this tissue. Although enzyme activity was not particularly high in the hepatopancreas and gill, the high mRNA expression levels of both GSTs indicates that transcription of both was upregulated during reoxygenation. Highest GST enzyme activity in the muscle demonstrates that this tissue responds later to hypoxia stress.

In conclusion, we cloned full-length MnGST-1 and MnGST-2 cDNAs from *M. nipponense*, and analyzed their mRNA expression levels and the enzyme activities of the encoded proteins in response to hypoxia and reoxygenation. Our findings indicate that MnGST-1 and MnGST-2 are hypoxia-inducible factors in *M. nipponense*, and both participate in oxidative stress under hypoxic conditions. The results suggest that the sensitivity of the two GST genes hypoxia varies from tissue to tissue, and there were also differences in the response patterns in the same tissue following acute and chronic hypoxic treatments.

## 4. Materials and Methods

### 4.1. Experimental Animals and Hypoxic Treatment

Three hundred healthy adult oriental river prawns with a wet weight of 3.0 ± 0.5 g were obtained from the Freshwater Fisheries Research Centre (FFRC), Chinese Academy of Fisheries Science Breeding Farm, Wuxi, China (120°13′44″ E, 31°28′22″ N). The suitable growth temperature of *M. nipponense* is 18–30 °C, pH ≤ 9 [46,47]. All samples were transferred to laboratory breeding conditions and maintained in six 500 L tanks with aerated fresh water for one week so that they could acclimate to their new environment. Prawns were fed snail meat twice a day, and culture conditions were 21.0 ± 0.5 °C, pH 8.2 ± 0.08, 6.0 ± 0.2 mg/L DO, and 0.08–0.09 mg/L total ammonia-nitrogen, with a 10 h light/14 h dark photoperiod.

In the acute hypoxia challenge experiment, 200 prawns were randomly divided into two groups (in triplicate) and maintained in filtered fresh water in treatment tanks at 6.0 ± 0.2 mg O2 L^−1^ (normoxic conditions) or 2.0 ± 0.2 mg O2 L^−1^ (hypoxic conditions) for 0, 12, or 24 h by keeping the hypoxia condition by bubbling with N2 gas until the desired O2 concentrations were reached; oxygen levels were maintained by adding N2 gas when needed. The change of dissolved oxygen in water was monitored in real time by using portable dissolved oxygen meter. After hypoxia challenge, this group was restored to normoxic conditions for 12 or 24 h. All exposures were conducted in triplicate for both control and treatment groups. Hepatopancreas, gill and muscle tissue from six prawns at each time point was pooled, immediately frozen in liquid nitrogen, and stored at −80 °C.

In the chronic hypoxia challenge experiment, 180 prawns were randomly divided into three groups (in triplicate) and maintained in filtered fresh water in treatment tanks at 6.0 ± 0.2 mg O2 L^−1^ (normoxic conditions), 4.5 ± 0.2 mg O2 L^−1^ (moderate hypoxia), or 3.0 ± 0.2 mg O2 L^−1^ (severe hypoxia) by nitrogen-filled manipulation [23]. All exposures were conducted in triplicate for both control and treatment groups. Hepatopancreas, gill and muscle tissue from six prawns at each time point was pooled, immediately frozen in liquid nitrogen, and stored at −80 °C.

### 4.2. Cloning of MnGST-1 and MnGST-2 cDNAs

RNAiso Plus Reagent (TaKaRa, Kusatsu, Japan) was used to isolate total RNA from hepatopancreas tissue according to the manufacturer’s protocol. The reverse transcriptase M-MLV Kit (TaKaRa, Kusatsu, Japan) was used to synthesize first-strand cDNA, and 3′-rapid amplification of cDNA ends (RACE) was performed using a 3′-full RACE Core Set Ver. 2.0 Kit (TaKaRa, Kusatsu, Japan) to determine the 3′ ends of *MnGST-1* and *MnGST-2*. All primers used for cloning are listed in Table 1. Polymerase chain reaction (PCR) products were purified using a gel extraction kit (CWBIO, Beijing, China) and sequenced using an ABI3730 DNA analyzer (ABI, Tampa, FL, USA) after insertion into the PMD-18T vector (TaKaRa, Kusatsu, Japan).

### 4.3. Nucleotide Sequence and Bioinformatics Analyses

The ORF Finder program (available online: http://ncbi.nlm.nih.gov/gorf/gorf.html) was used to deduce amino acid sequences, and BLASTX and BLASTN programs (available online: http://www.ncbi.nlm.nih.gov/BLAST/) were used to analyze protein and nucleotide sequences, respectively. The Motif Scan program (available online: http://hits.isbsib.ch/cgibin/motifscan/) was used to analyze motifs, and multiple sequence alignment was performed using the DNAMAN 6.0 (Lynnon Biosoft, San Ramon, CA, USA). The neighbor-joining (NJ) method was employed to construct complete phylogenetic trees using Molecular Evolutionary Genetics Analysis software version 4.0 (available online: http://www.megasoftware.net/mega4/mega.html). Signal sequence prediction was carried out using the YinOYang program (available online: http://www.cbs.dtu.dk/services/YinOYang).

### 4.4. Quantitative Real-Time PCR (QPCR) Analysis of MnGST-1 and MnGST-2 Expression

The mRNA levels of *MnGST-1* and *MnGST-2* in different tissues and following different hypoxic treatments were measured by QPCR. cDNAs from different tissues and following different hypoxia treatments were synthesized from total DNA-free RNA (1 μg) using a Prime Script RT reagent kit (TaKaRa, Kusatsu, Japan) following the manufacturer’s instructions. Reactions were executed on a Bio-Rad iCycler i Q5 Real-Time PCR system (Bio-Rad, USA) using primers listed in Table 1. Reaction conditions were as previously described [48]. The relative quantification of target and reference genes was evaluated using the standard curve method, and the amplification efficiency and threshold were automatically generated by standard curves. To select an appropriate and stable reference gene, ACTB (β-actin), 18s RNA (18S ribosomal RNA) and L8 (ribosomal protein L8) were tested, and the relative stability measure (M) was calculated by Ge Norm (available online: http://medgen.ugent.be/genorm/) [49]. *MnGST-1* and *MnGST-2* mRNA expression levels were calculated using the 2^−ΔΔCT^ method [50]. 

### 4.5. Semiquantitative Analysis of MnGST-1 and MnGST-2 Expression

Each sample was tested in triplicate in 25 μL reactions containing 1 μL cDNA (1 μg), 12.5 μL 2 × Es Taq MasterMix (dye), 1 μL of 10 μM gene-specific forward and reverse primers (Table 1), and 9.5 μL ddH_2_O. Samples were denatured for 3 min at 94 °C, followed by 30 cycles of denaturation at 94 °C for 30 s, primer annealing at 55 °C for 30 s, extension at 72 °C for 1 min, and a final extension at 72 °C for 10 min. PCR products were separated by 1.2% agarose gel electrophoresis to check the results of QPCR.

### 4.6. Enzyme Activity Assay

Gill, muscle and hepatopancreas samples were diluted to 10% homogenate (1:9 *m*/*v*) using 0.86% normal saline. The catalytic activities of *MnGST-1* and *MnGST-2* were measured spectrophotometrically using a GST Enzyme Activity Assay Kit (Njjcbio, Nanjing, China).

### 4.7. Statistical Analysis

All data are presented as mean ± standard error of the mean (SE; *n* = 3). Student’s *t*-tests were used to identify significant differences in *MnGST-1* and *MnGST-2* gene expression between controls and treatment samples using SPSS 20.0 software, and *p* ≤ 0.05 was considered significant. Single factor ANOVA (analysis of variance) was not only used to compare the significant differences of the same gene in different tissues, but also used to compare the differences of the same gene under different experimental treatment conditions. Single-factor ANOVA was performed by SPSS 20.0 software (SPSS, Chicago, IL, USA), and *p* ≤ 0.05 was considered significant.

## Figures and Tables

**Table 1:** List of primers used in this study.

Primer	Primer Sequence (5′-3′)
MnGST-1-F (Open reading frame)	CAGGTCTGTTATGCTGACTGCTA
MnGST-1-R (Open reading frame)	AAACTGTACCCACAGCGTTACAA
MnGST-2-F (Open reading frame)	CCGGGAGAATCCGAGAAGATTT
MnGST-2-R (Open reading frame)	AACCCTCATTTCTTCACTCGTCT
3′-MnGST-1 (3′ RACE out primer)	TTAGTGGCTTCAGTTTCCACCTT
3′-MnGST-1 (3′ RACE in primer)	GGCTTGGCTAGCAAGATGTAAAG
3′-MnGST-2 (3′ RACE out primer)	TGTCGAACACTACGTCAACATCA
3′-MnGST-2 (3′ RACE in primer)	TCAACTCTTGGATTGCAAAGTGC
MnGST-1-F (Real-time PCR primer)	ATTAGTGGCTTCAGTTTCCACCT
MnGST-1-R (Real-time PCR primer)	TGCCATTTTTCCAAATTCCTGGG
MnGST-2-F (Real-time PCR primer)	CGAAGCTCTGGAGTGGTTAGATG
MnGST-2-R (Real-time PCR primer)	AGTTGATGTTGACGTAGTGTTCG
β-Actin-F (Real-time PCR primer)	TATGCACTTCCTCATGCCAT
β-Actin-R (Real-time PCR primer)	AGGAGGCGGCAGTGGTCAT

**Table 2:** Mature peptide sequences of glutathione S-transferase (GST) family members.

Species	Gene Name	GenBank Accession Number
*Aethina tumida*	Predicted: glutathione S-transferase 1-like	XP_019870090.1
*Agrilus planipennis*	Predicted: glutathione S-transferase 1-like	XP_018323257.1
*Anopheles dirus*	glutathione S-transferase I	AAB41104.1
*Anoplophora glabripennis*	glutathione S-transferase 1-1	XP_023311254.1
*Antheraea pernyi*	glutathione S-transferase theta	ACB36909.1
*Callinectes sapidus*	glutathione S-transferase, partial	AJD14729.1
*Cephus cinctus*	glutathione-S-transferase 2	ARN17841.1
*Chilo suppressalis*	glutathione S-transferase δ 2	AKS40339.1
*Choristoneura fumiferana*	glutathione S-transferase 3	ABQ53631.1
*Cimex lectularius*	Predicted: glutathione S-transferase 1, isoform D-like	XP_014260234.1
*Cnaphalocrocis medinalis*	glutathione S-transferase δ 3	AIZ46898.1
*Cryptocercus punctulatus*	glutathione S-transferase d1	AFK49803.1
*Culicoides variipennis*	glutathione S transferase-1	AAB94639.1
*Delia antiqua*	glutathione S-transferase δ 1	ALF04571.1
*Dendroctonus ponderosae*	Predicted: glutathione S-transferase 1-like	XP_019755130.1
*Episyrphus balteatus*	putative glutahione S-transferase, δ class	CAH58743.1
*Eriocheir sinensis*	δ glutathione S-transferase GST	ACT78699.1
*Fenneropenaeus chinensis*	Delta class glutathione S-transferase	ANH58178.1
*Hyalella Azteca*	Predicted: glutathione S-transferase 1-like	XP_018013809.1
*Hyalella Azteca*	Predicted: glutathione S-transferase 1-like	XP_018010119.1
*Hyalella Azteca*	Predicted: glutathione S-transferase 1-like	XP_018007422.1
*Leptinotarsa decemlineata*	putative glutathione S-transferase δ class member 3	APX61027.1
*Leptinotarsa decemlineata*	glutathione S-transferase 1-like	XP_023021064.1
*Locusta migratoria*	glutathione S-transferase δ	ADR30117.1
*Macrobrachium nipponense*	MnGST-1	MG787172
*Macrobrachium nipponense*	MnGST-2	MG787173
*Macrobrachium rosenbergii*	GST- δ	CCQ19301.1
*Mayetiola destructor*	δ GST	ABG56084.1
*Palaemon carinicauda*	glutathione S-transferase	AGZ89666.1
*Periplaneta americana*	glutathione S transferase class δ variant 1	AEV23867.1
*Sogatella furcifea*	glutathione S-transferase D2	AFJ75818.1
*Stomoxys calcitrans*	Predicted: glutathione S-transferase 1-1	XP_013101447.1
*Tenebrio molitor*	glutathione S-transferase δ	AIL23531.1
*Tribolium castaneum*	Predicted: glutathione S-transferase 1, isoform D-like isoform X1	XP_974273.1
*Zootermopsis nevadensis*	glutathione S-transferase 1-1-like	XP_021941264.1

## Abbreviations

QPCR	Quantitative real-time reverse transcription PCR
ORF	Open reading frame
mRNA	Messenger RNA
*M. nipponense*	*Macrobrachium nipponense*
GST	Glutathione-S-transferase
DO	Dissolved oxygen
ROS	Reactive oxygen species
SOD	Superoxide dismutase
CAT	Catalase
GPx	Glutathione peroxidase
cDNA	Complementary DNA
FFRC	Freshwater Fisheries Research Centre
NJ	Neighbor-joining
